# Crystal structure of *catena*-poly[di­ammonium [di-μ-oxalato-cuprate(II)]]

**DOI:** 10.1107/S2056989016017631

**Published:** 2016-11-10

**Authors:** Diane A. Dickie, Richard A. Kemp

**Affiliations:** a Department of Chemistry and Chemical Biology, MSC03 2060, 1 University of New Mexico, Albuquerque, NM 87131, USA; b Advanced Materials Laboratory, Sandia National Laboratories, 1001 University Blvd SE, Albuquerque, NM 87106, USA

**Keywords:** crystal structure, copper, oxalate, hydrogen bonding, ammonium

## Abstract

The structure of the title compound, {(NH_4_)_2_[Cu(C_2_O_4_)_2_]}_*n*_, at 100 K has monoclinic (*P*2_1_/*c*) symmetry with the Cu^II^ atom on an inversion center. The compound has a polymeric structure due to long Cu⋯O inter­actions which create [Cu(C_2_O_4_)_2_] chains along the *a* axis. The structure also displays inter­molecular N—H⋯O hydrogen bonding, which links these chains into a three-dimensional network.

## Abstract 2

The polymeric title compound was formed unexpectedly from the reaction of bis­(diiso­propyl­phosphan­yl)amine with copper oxalate hemihydrate. It shows extensive N—H⋯O hydrogen bonding, as well as long Cu⋯O inter­actions.

## Chemical context   

Metal oxalate salts are ubiquitous in nature (Baran, 2014 ▸) and are also of great inter­est to synthetic chemists and materials scientists because they often display unusual magnetic and conductive properties (Nenwa *et al.*, 2015 ▸; Robinson *et al.*, 2015 ▸; Zhang *et al.*, 2012 ▸; Clemente-León *et al.*, 2011 ▸; Gruselle *et al.*, 2006 ▸). Other areas of study for metal oxalates include, but are not limited to, metallogels (Feldner *et al.*, 2016 ▸), coordination polymers and networks (Guo *et al.*, 2016 ▸; Mizzi & LaDuca, 2016 ▸; Yeşilel *et al.*, 2010 ▸), and precursors for nanomaterials and metallic inks (Yadav *et al.*, 2013 ▸; Cheng *et al.*, 2016 ▸). The properties of metal oxalates are often tuned by using a combination of different cations. These may be simply metal cations, but often they are more complex, such as quaternary nitro­gen cations. Surprisingly, the structure of the simplest of the (N*R*_4_)_2_[Cu(C_2_O_4_)_2_] family, (NH_4_)_2_[Cu(C_2_O_4_)_2_], has not previously been reported.
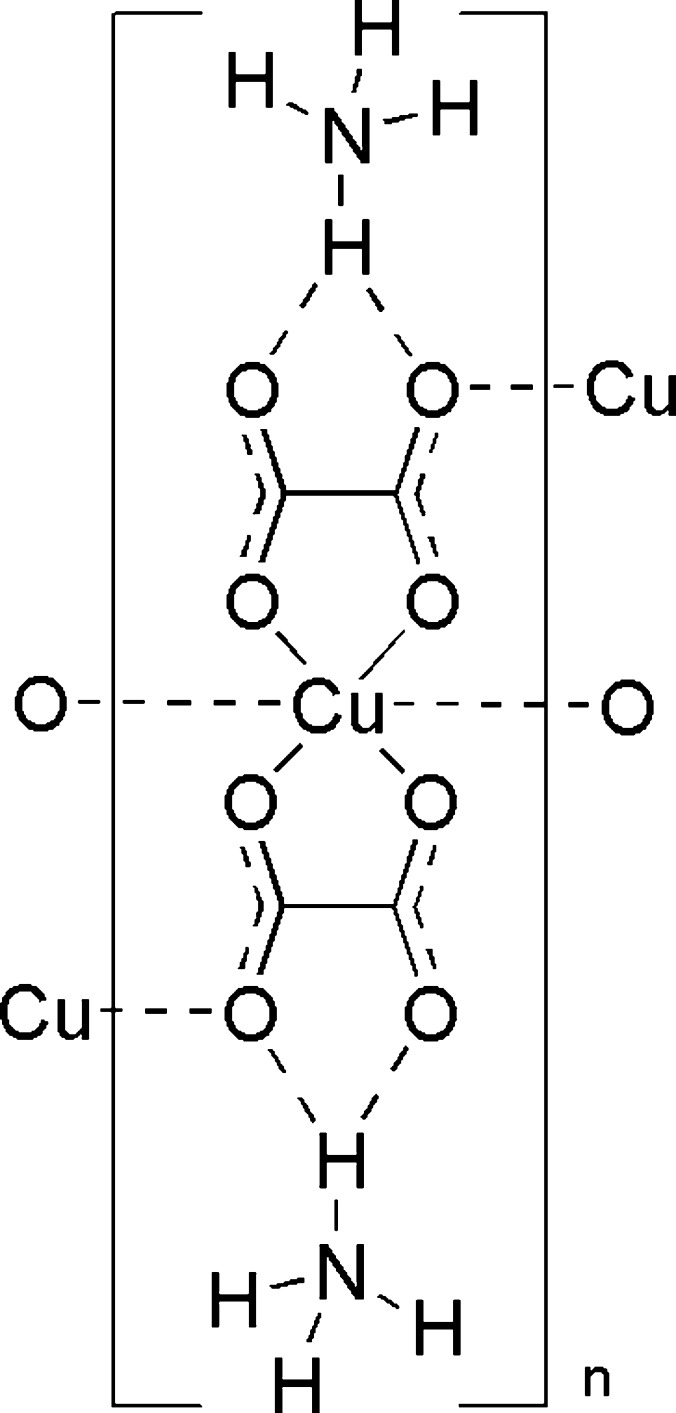


## Structural commentary   

**Figure 1:**
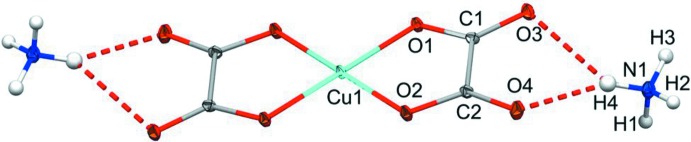
The mol­ecular structure of the title compound, with non-H atoms shown as displacement ellipsoids at the 50% probability level.

The title compound crystallizes in the monoclinic space group *P*2_1_/*c* with the copper atom on an inversion center (Fig. 1 ▸). As is true for all but one copper oxalate complex (Gu & Xue, 2007 ▸) found in the CSD (Version 5.37, May 2016 update; Groom *et al.*, 2016 ▸), the copper atom is chelated by oxygen atoms from the adjacent carbon atoms to form a five membered ring, rather than oxygen atoms from the same carbon. The coordination environment of copper is nearly perfectly square planar, with the O1—Cu1—O2 bond angle measuring 85.44 (3)° within the asymmetric unit, and 94.56 (3)° across the inversion center. Within the plane of the oxalate ligand, O1 and O2 form bonds to Cu1 measuring 1.9326 (7) and 1.9301 (7) Å, respectively. O3 inter­acts weakly, at a distance of 2.7057 (8) Å, with the symmetry-related Cu atoms above and below the ligand plane, giving an elongated octa­hedron. O4 has no bonding inter­actions with Cu, but does engage in hydrogen bonding with the ammonium cation (see below). The different ways that the oxygen atoms do or do not inter­act with copper is reflected in the C—O bonds. The two oxygen atoms that are strongly bound to copper, O1 and O2, have slightly longer bonds to carbon of 1.2798 (11) and 1.2895 (12) Å for C1—O1 and C2—O2, respectively. The weakly inter­acting O3 and non-bonded O4 have shorter C—O bonds of 1.2355 (12) and 1.2249 (12) Å for C1—O3 and C2—O4, respectively.

## Supra­molecular features   

**Figure 2:**
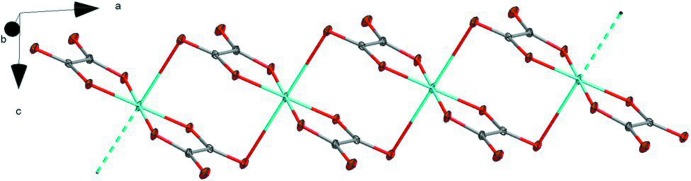
A single chain of the copper oxalate complex with the ammonium cation omitted.

**Figure 3:**
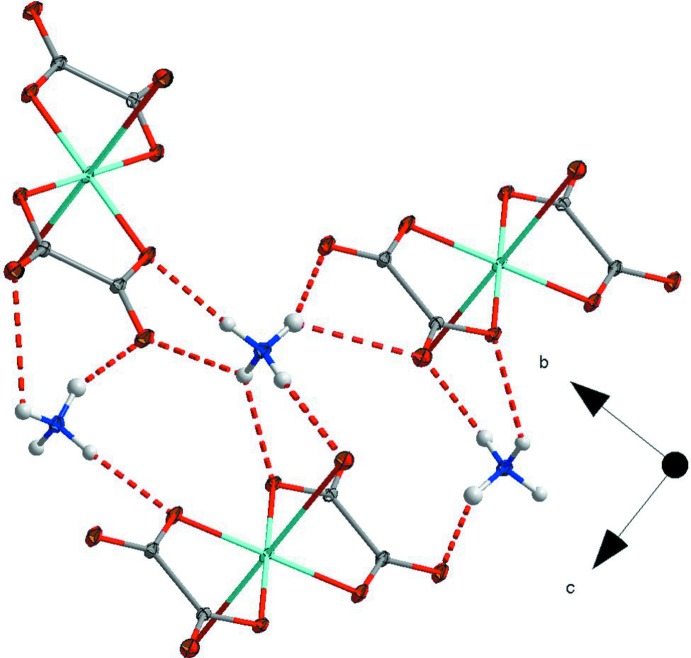
Packing viewed along the *a* axis, showing the polymeric complex formed by hydrogen bonding between the ammonium cations and oxalate ligands.

As noted above, the coordination sphere of the copper atoms is completed by a long inter­action of 2.7057 (8) Å between O3 and Cu1 in the planes above and below the ligand, giving rise to polymeric copper oxalate chains along the *a* axis (Fig. 2 ▸). These chains do not inter­act directly with one another. Instead, they are linked into a three-dimensional network by partly bifurcated N—H⋯O hydrogen bonds (Fig. 3 ▸) between all four protons of the ammonium cation and the oxalate oxygen atoms indicated by the symmetry operations in Table 1 ▸.

## Database survey   

There are three published reports of hydrated ammonium copper oxalate but to the best of our knowledge, the anhydrous title compound has not been reported previously. The earliest report, for (NH_4_)_4*n*_[Cu_2_(C_2_O_4_)_4_(H_2_O)_2_]_*n*_·2*n*H_2_O (Viswamitra, 1962 ▸) was re­inter­preted (Novosad *et al.*, 2000 ▸) as a polymeric complex with the repeat unit consisting of two [Cu(C_2_O_4_)_2_] moieties. One copper atom forms long Cu—O bonds to the next unit, similar to the way in which the title compound forms its chains, while the other is capped by two water mol­ecules. A different hydrate, (NH_4_)_8_[Cu_4_(C_2_O_4_)_8_(H_2_O)_2_·4H_2_O, has also been reported (Kadir *et al.*, 2006 ▸), but it does not feature chains of polymeric copper oxalate. Instead, it exists as a discrete water-capped tetra­mer. In each case, these hydrates also display hydrogen bonding between the oxalate, ammonium and water molecules. Besides the simple hydrates, structures of ammonium copper oxalates with polyoxidometalates based on tungsten (Reinoso *et al.*, 2005 ▸, 2007 ▸) or molybdenum (Li *et al.*, 2011 ▸) have been reported.

## Synthesis and crystallization   

A solution of bis­(diiso­propyl­phosphan­yl)amine (0.25 g, 1.0 mmol) in 1 mL MeOH was added to a slurry of copper(II) oxalate hemihydrate (0.15 g, 1.0 mmol) in 1 mL MeOH. The mixture was heated to reflux for 5 min and then allowed to cool to room temperature. After three days, the blue supernatant solution was deca­nted from an insoluble powder and cooled to 248 K. Block-like blue crystals of the title compound were isolated after six weeks. The mechanism by which bis(diiso­propyl­phosphan­yl)amine decomposes into ammonium is under investigation.

## Refinement   

Crystal data, data collection and structure refinement details are summarized in Table 2 ▸.

## Supplementary Material

Crystal structure: contains datablock(s) global, I. DOI: 10.1107/S2056989016017631/pj2038sup1.cif

Structure factors: contains datablock(s) I. DOI: 10.1107/S2056989016017631/pj2038Isup2.hkl

Supporting information file. DOI: 10.1107/S2056989016017631/pj2038Isup3.cdx

CCDC reference: 1515161

Additional supporting information: crystallographic information; 3D view; checkCIF report

## Figures and Tables

**Table 1:** Hydrogen-bond geometry (Å, °)

*D*—H⋯*A*	*D*—H	H⋯*A*	*D*⋯*A*	*D*—H⋯*A*
N1—H1⋯O1^i^	0.824 (17)	2.230 (17)	2.8667 (11)	134.2 (16)
N1—H1⋯O4^ii^	0.824 (17)	2.475 (17)	3.0503 (12)	127.8 (15)
N1—H2⋯O2^iii^	0.865 (18)	1.991 (18)	2.8507 (11)	172.5 (16)
N1—H3⋯O3^iv^	0.854 (19)	2.053 (19)	2.8884 (11)	165.7 (17)
N1—H4⋯O3	0.85 (2)	2.43 (2)	3.0015 (11)	124.7 (16)
N1—H4⋯O4	0.85 (2)	2.04 (2)	2.8330 (12)	155.1 (18)

Symmetry codes: (i) 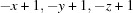; (ii) 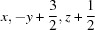; (iii) 
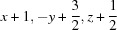
; (iv) 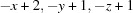.

**Table 2:** Experimental details

Crystal data	
Chemical formula	(NH_4_)_2_[Cu(C_2_O_4_)_2_]
*M*_r_	275.66
Crystal system, space group	Monoclinic, *P*2_1_/*c*
Temperature (K)	100
*a*, *b*, *c* (Å)	4.8564 (2), 13.5188 (5), 6.7205 (3)
β (°)	96.992 (2)
*V* (Å^3^)	437.94 (3)
*Z*	2
Radiation type	Mo *K*α
μ (mm^−1^)	2.53
Crystal size (mm)	0.43 × 0.28 × 0.28

Data collection	
Diffractometer	Bruker Kappa APEXII CCD
Absorption correction	Multi-scan (*SADABS*; Bruker, 2015 ▸)
*T*_min_, *T*_max_	0.41, 0.54
No. of measured, independent and observed [*I* > 2σ(*I*)] reflections	8277, 1672, 1537
*R*_int_	0.017
(sin θ/λ)_max_ (Å^−1^)	0.769

Refinement	
*R*[*F* ^2^ > 2σ(*F* ^2^)], *wR*(*F* ^2^), *S*	0.018, 0.051, 1.08
No. of reflections	1672
No. of parameters	86
H-atom treatment	All H-atom parameters refined
Δρ_max_, Δρ_min_ (e Å^−3^)	0.37, −0.51

Computer programs: *BIS* and *SAINT* (Bruker, 2015 ▸), *SHELXT* (Sheldrick, 2015*a* ▸), *SHELXL2014* (Sheldrick, 2015*b* ▸), *DIAMOND* (Brandenburg, 1999 ▸) and *publCIF* (Westrip, 2010 ▸).

## supplementary crystallographic information

### Crystal data

**Table:**

2(H_4_N^+^)·C_4_CuO_8_^2^^−^	*F*(000) = 278
*M**_r_* = 275.66	*D*_x_ = 2.090 Mg m^−^^3^
Monoclinic, *P*2_1_/*c*	Mo *K*α radiation, λ = 0.71073 Å
*a* = 4.8564 (2) Å	Cell parameters from 5449 reflections
*b* = 13.5188 (5) Å	θ = 3.0–33.1°
*c* = 6.7205 (3) Å	µ = 2.53 mm^−^^1^
β = 96.992 (2)°	*T* = 100 K
*V* = 437.94 (3) Å^3^	Block, blue
*Z* = 2	0.43 × 0.28 × 0.28 mm

### Data collection

**Table:**

Bruker Kappa APEXII CCD diffractometer	1672 independent reflections
Radiation source: fine-focus tube	1537 reflections with *I* > 2σ(*I*)
Graphite monochromator	*R*_int_ = 0.017
Detector resolution: 8.3333 pixels mm^-1^	θ_max_ = 33.2°, θ_min_ = 3.0°
φ and ω scans	*h* = −7→7
Absorption correction: multi-scan (SADABS; Bruker, 2015)	*k* = −20→18
*T*_min_ = 0.41, *T*_max_ = 0.54	*l* = −9→10
8277 measured reflections	

### Refinement

**Table:**

Refinement on *F*^2^	Primary atom site location: structure-invariant direct methods
Least-squares matrix: full	Hydrogen site location: difference Fourier map
*R*[*F*^2^ > 2σ(*F*^2^)] = 0.018	All H-atom parameters refined
*wR*(*F*^2^) = 0.051	*w* = 1/[σ^2^(*F*_o_^2^) + (0.0263*P*)^2^ + 0.1971*P*] where *P* = (*F*_o_^2^ + 2*F*_c_^2^)/3
*S* = 1.08	(Δ/σ)_max_ = 0.001
1672 reflections	Δρ_max_ = 0.37 e Å^−^^3^
86 parameters	Δρ_min_ = −0.51 e Å^−^^3^
0 restraints	

### Special details

**Table:**

Geometry. All esds (except the esd in the dihedral angle between two l.s. planes) are estimated using the full covariance matrix. The cell esds are taken into account individually in the estimation of esds in distances, angles and torsion angles; correlations between esds in cell parameters are only used when they are defined by crystal symmetry. An approximate (isotropic) treatment of cell esds is used for estimating esds involving l.s. planes.

### Fractional atomic coordinates and isotropic or equivalent isotropic displacement parameters (Å^2^)

**Table:**

	*x*	*y*	*z*	*U*_iso_*/*U*_eq_	
Cu1	0	0.5	0	0.00661 (5)	
O1	0.32614 (14)	0.44779 (5)	0.15885 (10)	0.00795 (12)	
O2	0.15437 (14)	0.62834 (5)	0.07337 (11)	0.00968 (13)	
O3	0.72392 (16)	0.50253 (5)	0.32622 (12)	0.01101 (14)	
O4	0.52948 (15)	0.69388 (5)	0.25066 (12)	0.01245 (14)	
C1	0.4930 (2)	0.51573 (7)	0.23064 (14)	0.00716 (15)	
C2	0.39072 (19)	0.62308 (7)	0.18343 (14)	0.00800 (15)	
N1	0.95132 (18)	0.67395 (6)	0.57854 (13)	0.00966 (14)	
H1	0.840 (3)	0.6723 (13)	0.662 (3)	0.020 (4)*	
H2	1.027 (3)	0.7319 (13)	0.582 (3)	0.019 (4)*	
H3	1.072 (4)	0.6285 (14)	0.607 (3)	0.023 (4)*	
H4	0.849 (4)	0.6643 (14)	0.468 (3)	0.029 (5)*	

### Atomic displacement parameters (Å^2^)

**Table:**

	*U*^11^	*U*^22^	*U*^33^	*U*^12^	*U*^13^	*U*^23^
Cu1	0.00454 (8)	0.00426 (8)	0.01054 (8)	−0.00002 (5)	−0.00113 (5)	0.00048 (5)
O1	0.0065 (3)	0.0063 (3)	0.0106 (3)	0.0002 (2)	−0.0011 (2)	0.0008 (2)
O2	0.0068 (3)	0.0060 (3)	0.0153 (3)	0.0005 (2)	−0.0026 (2)	−0.0002 (2)
O3	0.0084 (3)	0.0099 (3)	0.0136 (3)	0.0011 (2)	−0.0033 (2)	−0.0005 (2)
O4	0.0125 (3)	0.0075 (3)	0.0160 (3)	−0.0016 (2)	−0.0035 (2)	−0.0014 (2)
C1	0.0071 (3)	0.0064 (3)	0.0081 (3)	0.0007 (3)	0.0013 (3)	0.0003 (3)
C2	0.0074 (3)	0.0070 (4)	0.0094 (3)	0.0009 (3)	0.0005 (3)	0.0000 (3)
N1	0.0100 (3)	0.0075 (3)	0.0110 (3)	−0.0017 (3)	−0.0007 (3)	0.0004 (3)

### Geometric parameters (Å, º)

**Table:**

Cu1—O2^i^	1.9301 (7)	O4—C2	1.2249 (12)
Cu1—O2	1.9301 (7)	C1—C2	1.5541 (13)
Cu1—O1^i^	1.9326 (7)	N1—H1	0.824 (17)
Cu1—O1	1.9326 (7)	N1—H2	0.865 (18)
O1—C1	1.2798 (11)	N1—H3	0.854 (19)
O2—C2	1.2895 (12)	N1—H4	0.85 (2)
O3—C1	1.2355 (12)		
			
O2^i^—Cu1—O2	180.00 (4)	O1—C1—C2	114.91 (8)
O2^i^—Cu1—O1^i^	85.44 (3)	O4—C2—O2	125.44 (9)
O2—Cu1—O1^i^	94.56 (3)	O4—C2—C1	120.44 (8)
O2^i^—Cu1—O1	94.57 (3)	O2—C2—C1	114.12 (8)
O2—Cu1—O1	85.43 (3)	H1—N1—H2	108.8 (16)
O1^i^—Cu1—O1	180.0	H1—N1—H3	108.4 (16)
C1—O1—Cu1	112.64 (6)	H2—N1—H3	111.5 (16)
C2—O2—Cu1	112.83 (6)	H1—N1—H4	103.3 (16)
O3—C1—O1	125.83 (9)	H2—N1—H4	111.1 (16)
O3—C1—C2	119.23 (8)	H3—N1—H4	113.3 (17)
			
Cu1—O1—C1—O3	−175.08 (8)	O3—C1—C2—O4	−4.51 (14)
Cu1—O1—C1—C2	2.83 (9)	O1—C1—C2—O4	177.43 (9)
Cu1—O2—C2—O4	−179.39 (8)	O3—C1—C2—O2	176.24 (9)
Cu1—O2—C2—C1	−0.18 (10)	O1—C1—C2—O2	−1.82 (12)

Symmetry code: (i) −*x*, −*y*+1, −*z*.

### Hydrogen-bond geometry (Å, º)

**Table:**

*D*—H···*A*	*D*—H	H···*A*	*D*···*A*	*D*—H···*A*
N1—H1···O1^ii^	0.824 (17)	2.230 (17)	2.8667 (11)	134.2 (16)
N1—H1···O4^iii^	0.824 (17)	2.475 (17)	3.0503 (12)	127.8 (15)
N1—H2···O2^iv^	0.865 (18)	1.991 (18)	2.8507 (11)	172.5 (16)
N1—H3···O3^v^	0.854 (19)	2.053 (19)	2.8884 (11)	165.7 (17)
N1—H4···O3	0.85 (2)	2.43 (2)	3.0015 (11)	124.7 (16)
N1—H4···O4	0.85 (2)	2.04 (2)	2.8330 (12)	155.1 (18)

Symmetry codes: (ii) −*x*+1, −*y*+1, −*z*+1; (iii) *x*, −*y*+3/2, *z*+1/2; (iv) *x*+1, −*y*+3/2, *z*+1/2; (v) −*x*+2, −*y*+1, −*z*+1.
